# Dual-Energy Lung Perfusion in Portal Venous Phase CT—A Comparison with the Pulmonary Arterial Phase

**DOI:** 10.3390/diagnostics11111989

**Published:** 2021-10-26

**Authors:** Isabelle Praël, Wolfgang Wuest, Rafael Heiss, Marco Wiesmueller, Markus Kopp, Michael Uder, Matthias S. May

**Affiliations:** 1 Department of Radiology, University Hospital Erlangen, 91054 Erlangen, Germany; isabelle.prael@outlook.com (I.P.); rafael.heiss@uk-erlangen.de (R.H.); marco.wiesmueller@uk-erlangen.de (M.W.); markus.kopp@uk-erlangen.de (M.K.); michael.uder@uk-erlangen.de (M.U.); 2 Faculty of Medicine, Friedrich-Alexander-Universität Erlangen-Nürnberg (FAU), 91054 Erlangen, Germany; 3 Imaging Science Institute Erlangen, 91054 Erlangen, Germany; wolfgang.wuest@martha-maria.de; 4 Hospital Martha-Maria, 90491 Nürnberg, Germany

**Keywords:** dual-energy computed tomography, lung perfusion, portal venous

## Abstract

Pulmonary arterial dual-energy (aDE) CT is an established technique for evaluating pulmonary perfusion blood volume (PBV). As DECT protocols are increasingly used for thoraco-abdominal CT, this study assessed image quality and clinical findings in portal–venous phase dual-energy (vDE) CT and compared it to aDE. In 95 patients, vDE-CT was performed using a dual-source scanner (70/Sn150 kV, 560/140 ref.mAs). Pulmonary triggered aDE-CT served as reference (*n* = 94). PBV was reconstructed using a dedicated algorithm. Mean relative attenuation was measured in the pulmonary trunk, aorta, and segmented lung parenchyma. A distribution ratio (DL) between vessels and parenchyma was calculated to assess the iodine uptake of the lung parenchyma. Subjective overall diagnostic image quality was assessed for PBV images on a five-point Likert scale. Image artifacts were classified into five groups based on scale rating and compared between vDE and aDE. Pathological findings were correlated with the anatomical image datasets. Mean relative attenuation of the lung parenchyma was comparable in both groups (vDE: 23 ± 6 HU and aDE: 22 ± 7 HU), but significantly lower in the vessels of vDE. Therefore, iodine uptake of the lung parenchyma was significantly higher in vDE (DL: 10% vs. 8%, *p* < 0.01). The subjective overall image quality of the PBV images was comparable (*p* = 0.5). Rotation and streak artifacts were found in most of the patients (>86%, both *p* > 0.6). Dual-source artifacts were found in only a few patients in both groups (vDE 5%, aDE 7%, *p* = 0.5). Recess and subpleural artifacts were increased in vDE (vDE 53/27%, aDE 24/7%, both *p* < 0.001). Pathological findings were found in 19% of the vDE patients and 59% of the aDE patients. Comparable objective and subjective image quality of lung perfusion can be obtained in vDE and aDE. Iodine uptake of the lung parenchyma is increased in vDE compared to aDE, suggesting an interstitial pooling effect. Knowledge of the different appearances of artifacts will aid in the interpretation of the images. Additional clinical information about the lung parenchyma can be provided by PBV evaluation in vDE.

## 1. Introduction

Dual-energy CT (DECT) is a well-established and often used technique in the field of thoracic imaging. The combination of functional and anatomic images allows visualization of the perfusion blood volume (PBV) of the lung parenchyma [1]. Many previous studies have shown that DECT in the arterial phase (aDE) can achieve a solid image quality and be successfully used for the detection and evaluation of pulmonary thromboembolism (PTE) and diagnosis of pulmonary hypertension [2,3,4].

DECT protocols are also increasingly used for thoraco-abdominal CT in the portal–venous contrast agent phase without increasing patient radiation dose compared to single-energy CT [5,6]. However, there is no information about PBV reconstructions from these data in the literature. Studies on dual-phase PBV recently presented interesting results about the dynamic effects of contrast enhancement in the lung parenchyma. Hong et al. [7] acquired images at early aDE 2 s after the bolus arrival and at delayed aDE 20 s after the early aDE acquisition; segments with perfusion and filling defects in patients with chronic PTE had significantly increased iodine-related-attenuation in delayed-phase images, possibly resulting from increased systemic collateral inflow. They, furthermore, stated that double phase DECT could be used for differentiation between acute and chronic PTE. Koike et al. [8] also quantified lung PBV in patients with chronic PTE in early and late aDE with fixed timing of 14 and 40 s after contrast injection and concluded that global perfusion from systemic collaterals can be depicted. We therefore speculated that PBV reconstructions from vDE provide different functional information compared to aDE, making it difficult for radiologists to evaluate these images [9]. Moreover, potential image artifacts have only been quantified in aDE. Our aim was therefore to assess physiology, image quality, and incidental findings of lung PBV images in vDE from patients who underwent thoraco-abdominal staging CT and to compare these to aDE, following the null-hypothesis that there are no differences.

## 2. Materials and Methods

### 2.1. Patients

**Figure 1:**
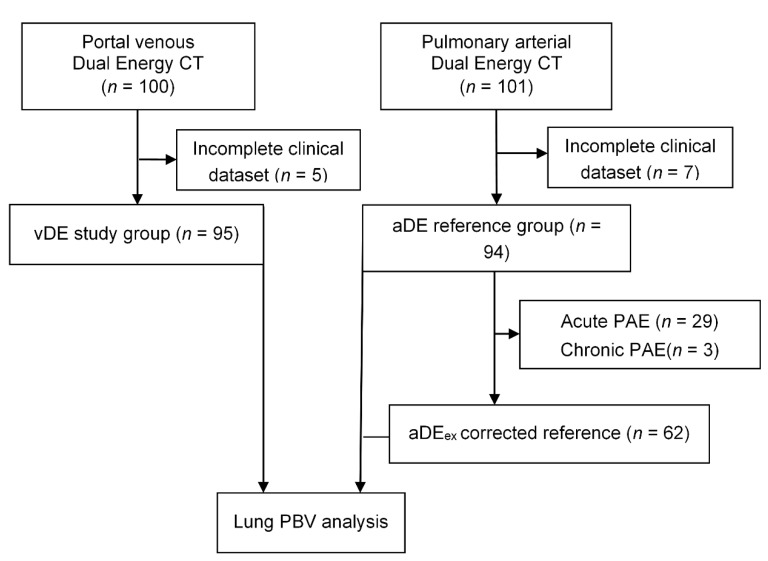
Flowchart of study design (vDEportal-venous thoraco-abdominal Dual Energy CT, aDE—pulmonary arterial Dual Energy CT, PAE—pulmonary arterial embolism, aDEex—subgroup of aDE with exclusion of PAE patients, PBV—perfusion blood volume).

A collective of 100 consecutive patients who underwent thoraco-abdominal staging CT in portal venous phase from May 2015 to October 2015 was retrospectively selected for evaluation in the vDE study group. All CT images were acquired using a DECT protocol on a third-generation Dual Source CT (Somatom Force, Siemens Healthcare GmbH, Forchheim, Germany). For the reference group, 101 consecutive patients, who underwent pulmonary arterial DECT on the same CT-scanner from June 2015 to August 2016, were retrospectively selected for aDE evaluation. Inclusion criteria were age over 18 years, use of contrast-enhanced protocols, and complete coverage of the lungs in z-direction for both groups. Patients with incomplete clinical data (weight, height) were excluded (vDE *n* = 5, aDE *n* = 7; see Figure 1). An additional subgroup of aDE (aDEex *n* = 62) was formed by the exclusion of all patients with findings of acute or chronic PAE, to correct for selection bias. The study was performed using protocols that were approved by the institutional review board and complies with the Declaration of Helsinki. Written informed consent for study participation was waived due to the retrospective study design.

### 2.2. Dual Energy CT

All examinations were performed using a spiral technique that used the institutional reference protocols for pulmonary arterial DECT of the chest and portal venous thoraco-abdominal DECT. Scan parameters are shown in Table 1. Differences between the protocols are mainly due to contrary requirements, since speed is a major issue in pulmonary arterial timing and soft tissue homogeneity and spectral separation is a major issue in thoraco-abdominal imaging. For both groups, a tin prefiltration (Sn, 0.6 mm) was used for Tube B to increase the spectral separation of the DE dataset. Contrast medium (Iomeprol, Imeron350, Bracco, Milan, Italy) was injected automatically using a dual-head power injector (Accutron CT-D, Medtron, Saarbrücken, Germany). Contrast volume was 100 mL for vDE and 60 mL for aDE at a flow rate of 3 mL/s, both followed by a saline flush (30 mL NaCl). Scans were performed in craniocaudal direction and patients were placed in the supine position. The same inspiratory breathing command was given to both collectives prior to acquisition. A delay of 70 s was used for vDE, and bolus tracking was +8 s for aDE.

### 2.3. Image Reconstruction

**Figure 2:**
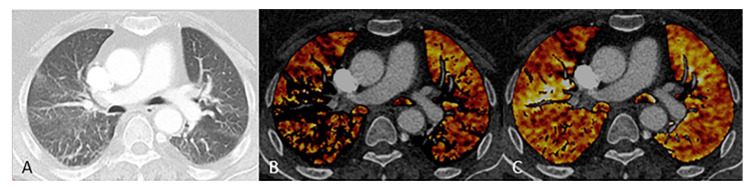
Technique of perfusion blood volume (PDV) image reconstruction: 66-year-old female from the reference group (aDE—pulmonary arterial Dual Energy CT) with chronic pulmonary arterial embolism. (**A**) conventional reconstruction, (**B**) incomplete lung segmentation due to increased density of the lung parenchyma above the upper threshold (−600 HU), and (**C**) full segmentation of the lung parenchyma after adapting the upper threshold to –300 HU. Both PBV images are displayed in the same window (width 80 HU, center 40 HU).

Standard anatomic reconstructions were performed on the CT system using a soft (Br40) and sharp reconstruction kernel (Br60) for thin (slice thickness 1.0 mm, increment 0.7 mm) and thick slices (5.0 mm/5.0 mm). DE reconstructions were calculated in thin slices (1.0 mm/0.7 mm) for the high and low energy datasets using a smooth quantitative kernel (Qr40). All series were archived in a picture archiving and communicating system. Lung PBV calculation was performed using a dedicated post-processing algorithm (Syngo.via, VB30, Siemens Healthcare GmbH, Forchheim, Germany) that automatically segments the lung parenchyma based on its attenuation in the CT-image. A low-pass filter was applied, and iodine enhancement in Hounsfield Units (HU) was calculated from the DECT data based on material decomposition theory [1]. The obtained iodine enhancement in the different parts of the lung was approximately proportional to the perfused blood volume (PBV) and served as a surrogate for it. Axial and coronal color-coded PBV-images were reconstructed as multi-planar reformations (5.0 mm/5.0 mm) using a narrow window (width 80, center 40) and were archived for further evaluation. Default settings were used for resolution (4 out of 10), iodine ratio (3.01) and segmentation thresholds (minimum −960 HU, maximum −600 HU) for all patients. Adapted upper thresholds (−300 HU) were used in case of incomplete lung segmentation due to increased density of the parenchyma (Figure 2).

### 2.4. Objective Image Evaluation

Total pulmonary volumes (VT) and mean relative attenuation value of the total lung parenchyma in HU (AT) were automatically calculated and recorded from the structured report of the DE algorithm. Mean relative attenuation in the ascending aorta (AA) and the pulmonary trunk (AP) were measured by manual region of interest definition (ROI). ROIs were positioned in the vessels to be as large as possible while thoroughly avoiding plaques and artifacts. AT was then referenced with the average of AA and AP to calculate a distribution ratio (DL) of contrast agent between the large vessels and the lung parenchyma:

Equation (1):

(1) D”L”=100%∗([2A]_T)/(A_A+A_P)

### 2.5. Subjective Image Evaluation

**Figure 3:**
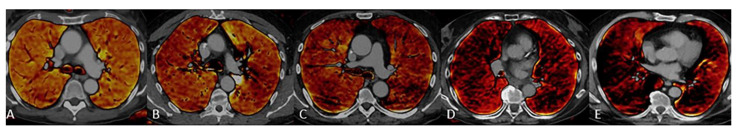
Examples for subjective overall diagnostic image quality of the perfusion blood volume (PBV) images: (**A**) excellent (Likert 1), (**B**) good (2), (**C**) adequate (3), (**D**) deficient (4), (**E**) poor (5). All images are displayed in the same window (width 80 HU, center 40 HU).

The overall subjective image quality of PBV maps was assessed on a five-point Likert scale by two independent radiologists with 9 and 10 years of experience (Figure 3): 1—excellent (homogeneous distribution, no artifacts, unequivocal delineation of pathologies); 2—good (mainly homogeneous distribution, few artifacts, clear delineation of pathologies); 3—adequate (in parts homogeneous distribution, several artifacts, vague delineation of pathologies); 4—deficient (mainly inhomogeneous distribution, excessive artifacts, doubtable delineation of pathologies); and 5—poor (mainly no iodine detection, artifacts not assignable, and pathologies). Subjective image quality results were additionally used as input for a subgroup analysis of AT to evaluate potential reliance. 

### 2.6. Artifacts

**Figure 4:**
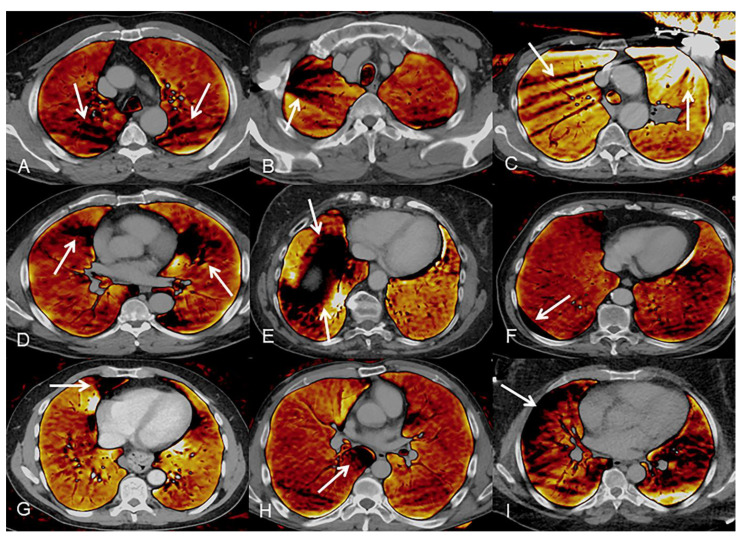
Examples of image artifacts in the perfusion blood volume (PBV) images: (**A**) streak artifacts along the spine, (**B**) streak artifacts around venous contrast agent inflow, (**C**) streak artifacts around metallic objects, (**D**) rotation artifacts around the heart, (**E**) rotation artifacts around the diaphragm, (**F**) Dual-Source artifacts, (**G**) anterior recess artifacts, (**H**) posterior recess artifacts, (**I**) subpleural artifacts.

Image artifacts in PBV were evaluated on a dichotomous scale (1—no artifact; 2—remarkable artifact) and classified into five groups with seven subgroups: streaks (spine, venous contrast inflow, and foreign objects), rotation (heart and diaphragm), dual source, recesses (anterior costo-mediastinal recess and posterior azygo-esophageal recess), and subpleural. Streak artifacts arise from beam hardening at the edge of structures with high attenuation and were mainly found around the spine, around metallic foreign objects, and at the slice of highly concentrated contrast media inflow via the subclavian and brachiocephalic veins or the vena cava superior. Rotation artifacts are attributed to the spiral acquisition technique and occur around objects with considerable density differences, like the heart or the liver dome surrounded by lung tissue. Dual-source artifacts occur if the lungs are not completely covered by both detectors of the scanner, which affects the field of views larger than 353 mm. Areas of reduced PBV were also systematically found in the anterior costo-mediastinal recesses of the lingula and the middle lobe, as well as the posterior azygo-esophageal recess. Subpleural artifacts describe a comparable effect of reduced PBV in the peripherally located subpleural area. Examples for all groups of artifacts are given in Figure 4.

### 2.7. Pathologic Findings

All increased or decreased PBV-findings that were not identified as artifacts were considered as pathologic perfusion. Correlating anatomic findings in the conventional lung reconstructions (Br60, lung window) were classified as decreased vascularization (e.g., acute PTE, oligemia), increased vascularization (e.g., chronic PTE, hyperemia), decreased density (e.g., emphysema), increased density (e.g., consolidation, atelectasis), or architectural distortion (e.g., postural anomalies, scar tissue). Findings without anatomic correlations had two main distribution patterns: lobar and anteroposterior. PBV images with poor overall image quality (Likert 5) were considered non-diagnostic and were not used for evaluation of pathological findings.

### 2.8. Statistics

All statistical analyses were performed using the software package Minitab 19 (Minitab Inc., State College, PA, USA). The normal distribution of the data was tested by the Kolmogorov–Smirnov test. Normally distributed data are presented as mean ± standard deviation. Objective image quality was compared between vDE and aDE/aDEex using a paired *t*-test. Subjective image quality was compared using the Mann–Whitney-U test. Subgroup analysis for AT was carried out using analysis of variance with pair-wise comparisons as proposed by Fisher. The significance level was defined as *p* < 0.05. Interrater agreement was assessed using Cohen’s kappa test; values > 0.61 were interpreted as substantial and >0.81 as almost perfect agreement, following Landis and Koch method [10].

## 3. Results

### 3.1. Patients

The vDE study group consisted of 70 male and 25 female patients with a mean age of 64 ± 13 years and a mean BMI of 25.8 ± 4.6 kg/m^2^. The aDE reference group consisted of 53 male and 41 females with a mean age of 66 ± 14 years and a mean BMI of 27.8 ± 5.8 kg/m^2^. A total of 29 patients with acute and 3 patients with chronic PTE were excluded in the corrected reference subgroup (aDEex).

### 3.2. Objective Image Quality

The results of the mean pulmonary volume (VT), the mean relative attenuation (AT/A/P), and the mean distribution ratio (DL) are given in Table 2 for vDE, aDE, and aDEex. The *p*-values are provided for the comparisons between vDE and aDE and aDEex. Mean pulmonary volumes were significantly lower in aDE. The mean relative attenuation in the large intrathoracic vessels (AA and AP) was significantly lower in vDE compared to aDE and aDEex (*p* < 0.03). However, AT was comparable between vDE and aDE, as well as aDEex (*p* = 0.87 and *p* = 0.84). Hence, DL was significantly increased in vDE (*p* = 0.001), compared to aDE and aDEex.

### 3.3. Subjective Image Quality

**Figure 5:**
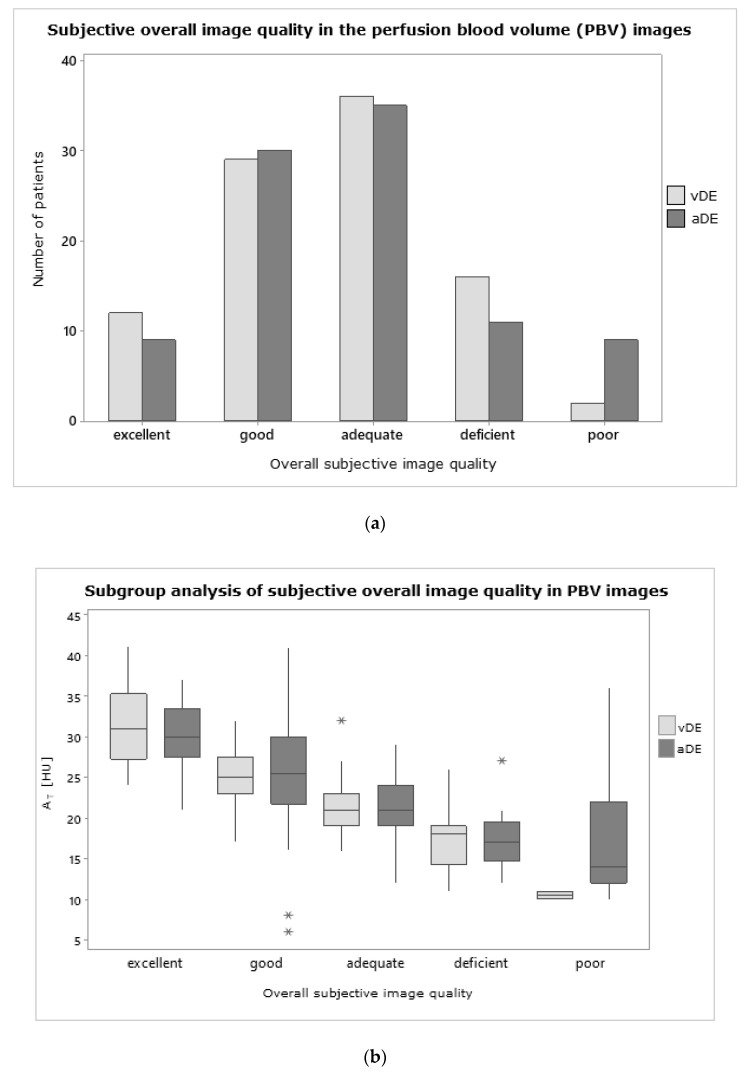
(**a**) Prevalence of subjective overall image quality in the perfusion blood volume (PBV) images compared between the study (vDE—portal-venous thoraco-abdominal Dual Energy CT) and the reference group (aDE—pulmonary arterial Dual Energy CT). (**b**) Subgroup analysis of the mean relative attenuation of the lung parenchyma (AT) in dependence of the subjective overall image quality in the PBV images. * *p* = 0.10.

The overall subjective image quality of PBV reconstructions was comparable between vDE and aDE; no statistically significant differences were noted (*p* = 0.52). The median rating was adequate (Likert 3) in both groups. Excellent image quality was slightly more frequent in vDE (12.6% vs. 9.6%), while poor ratings were more frequent in aDE (2.1% vs. 9.6%, Figure 5a). Overall, subjective image quality was also comparable between vDE and aDEex; no statistically significant differences were noted (*p* = 0.10). Interrater agreement was substantial for each group and parameter; mean kappa was 0.75. AT in patients with good subjective image quality ratings was higher than in patients with low subjective image quality ratings (Figure 5b). Differences between the subgroups were statistically significant for vDE (all *p* < 0.01). For aDE, no significance was found for the comparisons between Likert categories 1 and 2 (*p* = 0.054), as well as between Likert categories 3 to 5 (all *p* > 0.08).

### 3.4. Artifacts

The occurrence of image artifacts in PBV is illustrated in Table 3. The frequency of streak artifacts was comparable in both groups (*p* = 0.56), but with significantly more cases (both *p* <0.001) around the spine in vDE (86%, aDE 37%) and around the highly concentrated contrast media inflow in the venous vessels in aDE (75%, vDE 28%). Rotation artifacts were found in most cases in both groups (vDE: 94%, vDE: 89%, *p* = 0.57), while dual-source artifacts were seldom seen in either group (vDE: 5%, aDE: 7%, *p* = 0.54). Recess artifacts and subpleural artifacts were substantially more frequent (both *p* < 0.001) in vDE (53% and 27%) compared to aDE (24% and 7%). At least one artifact was found in every patient in vDE and aDE. The average of detected artifacts was 3.5 per patient in vDE and 2.9 in aDE.

### 3.5. Pathologic Findings

**Figure 6:**
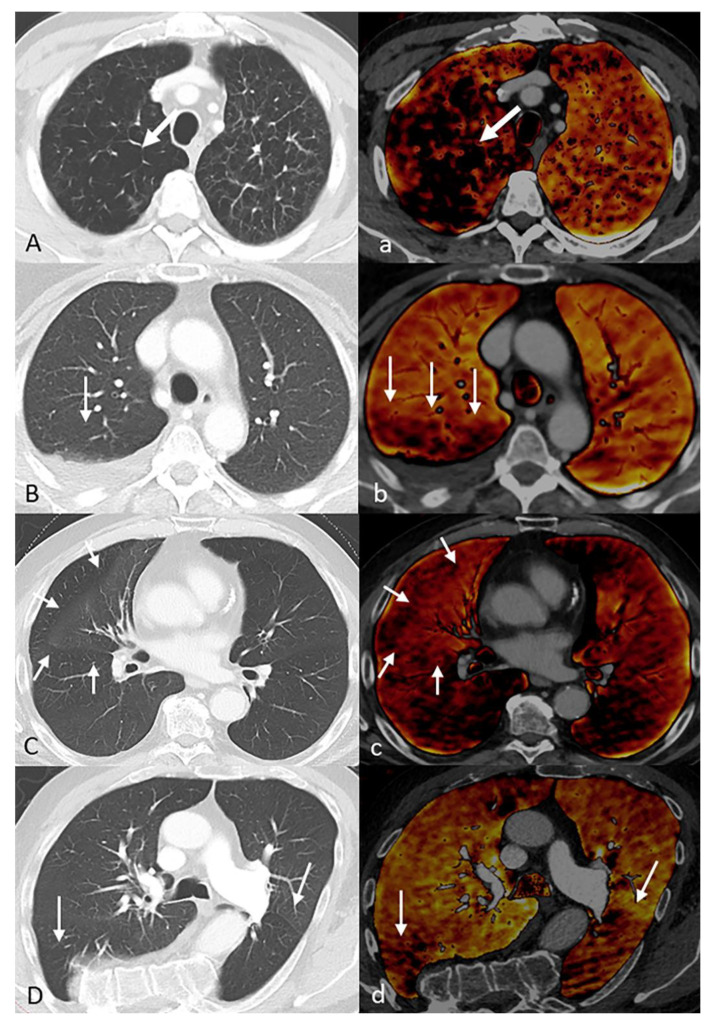
Examples of pathological findings in the perfusion blood volume (PBV) images (**a**–**d**) with correlating anatomic findings in the conventional lung reconstructions (**A**–**D**): (**A**/**a**)—57-year-old female patient from the study group (vDE—portal-venous thoraco-abdominal Dual Energy CT) with lung emphysema. Areas of reduced PBV match the bullae of the emphysema (decreased parenchyma density). (**B**/**b**)—67-year-old female patient (vDE) with right-sided pleural effusion and adjacent compression of the lung parenchyma. The area reduced PBV is much larger than the visible atelectasis in B (increased parenchyma density). (**C**/**c**)—69-year-old male patient (vDE) with hyperemia of the middle lobe. PBV proves increased iodine content and hence increased perfusion. The increased density in (**C**) could also be misinterpreted as hypoventilation (increased parenchyma density). (**D**/**d**)—77-year-old male patient (vDE) with severe scoliosis. Areas of reduced PBV can be seen in strongly deformed segments of the lung (architectural distortion).

**Figure 7:**
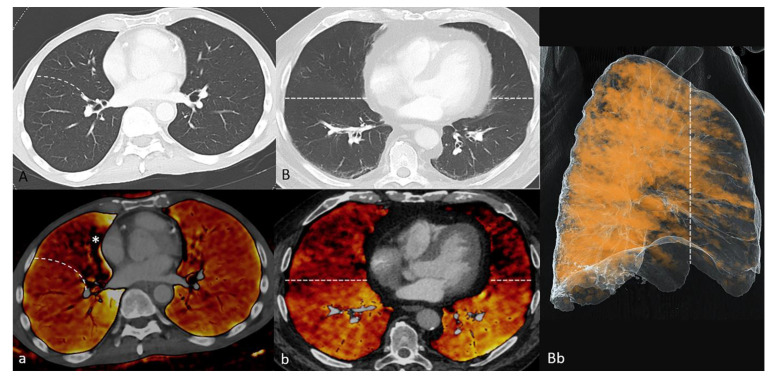
Examples of pathological findings in the perfusion blood volume (PBV) images (**a**,**b**) without anatomic correlates (**A**,**B**): (**A**/**a**)—50-year-old male patient from the study group (vDE—portal-venous thoraco-abdominal Dual Energy CT). An area of reduced PBV, limited to the middle lobe (oblique fissure: dashed white line), can be differentiated from a small rotation artifact around the heart (asterisk). (**B**/**b**/**Bb**) 79-year-old male patient from the reference group (aDE—pulmonary arterial Dual Energy CT). The PBV is reduced in the anterior parts of both lungs (in front of the dashed white line), without respecting fissural borders (anteroposterior gravity gradient).

PBV findings were identified in 18 vDE patients (19%) and in 51 aDE patients (59%, 23 in aDEex 37%). Findings with increased PBV were detected in three vDE and seven aDE patients. All others had decreased PBV. Pathologic correlates were found in the anatomic reconstructions for almost two-thirds of cases using vDE (61%) and three-quarters using aDE (76%). Lesions with decreased vascularization in the anatomical images were more frequently detected with aDE (48% of all patients, vDE 6%) and were always associated with pulmonary embolism in aDE. Increased vascularization was only detected with aDE (6%) and was always associated with increased PBV in chronic PTE. Decreased parenchyma density was always associated with reduced PBV in both groups. Increased parenchyma density was found in 10 patients and was associated with increased PBV in most cases (*n* = 7, 70%). Two patients in vDE and two patients in aDE showed decreased PBV in areas of architectural distortion. More than one-third of the findings in vDE (39%) and almost one-quarter in aDE (24%) had no anatomical correlate—All of the findings in vDE were linked to the lobar or segmental anatomy of the lungs. In contrast, a considerable part of the findings without morphological correlate in aDE had an anteroposterior distribution gradient (42%). Detailed results are shown in Table 4; examples are provided in Figure 6 and Figure 7.

## 4. Discussion

The mean relative attenuation as a surrogate for PBV in the lungs was comparable between aDE and vDE. The higher distribution ratio between the great vessels and the lung parenchyma in vDE suggests an accumulation effect of contrast agent in the interstitial space of the lungs, comparable to portal venous phase images of the liver or nephrogenic phase images of the kidneys. This is in line with the increased ratios found at 40 s (0.11) compared to 14 s (0.09) by Koike et al. [8]. In contrast to our study, Koike et al. used a ratio between tissue and pulmonary arterial uptake; the respective values from our data are 0.10 in vDE and 0.08 in aDE. This extends the current concept of single-phase lung imaging to a dynamic process [11].

Subjective image quality was comparable between PBV reconstructions in vDE and aDE. This correlates well with the measured mean relative attenuation of the lung parenchyma in both groups. However, the reason for the high inter-individual PBV variance remains unclear. Unfortunately, we were not able to correlate this to clinical parameters like pulmonary or cardiac function tests in our retrospective study design. Future prospective investigations would be needed to systematically investigate this finding.

The types of PBV artifacts found in our study are comparable to the report of Kang et al., who evaluated arterial PBV images obtained from a first-generation dual-source scanner and emphasized the importance of knowing the most common defects and artifacts for more accurate interpretation of aDE [9]. Frequencies of artifacts are comparable between aDE and vDE in our data, but the types are slightly different. Streak artifacts changed from more inflow artifacts in aDE, due to the early contrast timing, to more spine artifacts in vDE. We attribute this most likely to be due to an increased beam hardening effect, provoked by the lower main tube voltage (70 kV vs. 80 kV) in the portal venous phase protocol [12,13]. Future protocols could overcome these problems by using a tri-phasic injection protocol on one side and by increasing the tube voltage to 80 or 90 kV on the other [14,15]. Dual-source artifacts were seldom seen in either group. However, new automated positioning algorithms using a three-dimensional camera as is described for radiation dose reduction in the literature could also help to further avoid incomplete coverage in DE examinations [16]. More recess and subpleural artifacts were detected in vDE, without any physical explanation. Therefore, we speculate that these hypo-attenuations in portal venous PBV reconstructions may reflect truly decreased blood supply to these peripherally located regions. This complies with the findings of Felloni et al. who reported a median difference of nearly 10 HU between the mean level of attenuation in medullary and cortical lung zones [17].

Pathological findings were more frequent in the pulmonary arterial phase, even if PAE cases were excluded. We attribute this to a selection bias on the basis of clinically symptomatic patients with shortness of breath that were enrolled in the reference group and rather asymptomatic staging patients in the study group. This also correlates to the lower lung volumes measured in aDE. However, incidental findings in the portal venous phase could be found in every fifth patient, on average. It seems especially promising to retrospectively differentiate incidental morphologic changes in routine acquisitions, e.g., to evaluate the underlying disease in mosaic pattern attenuation alterations. Moreover, almost half of these findings could not be detected in the conventional anatomical reconstructions. The lobar or segmental distribution may be explained by restricted arterial supply, and the anterior-posterior gradient is in good agreement with reports about gravity-dependent lung perfusion in supine position [16,17].

Our study has several limitations. First, our retrospective study design comes along with some restrictions. The indication for a CT examination of the chest differed between our two patient-collectives, resulting in two different examination protocols. The lower tube voltage in vDE may be an explanation for the increased frequency of streak artifacts around the spine on one side, but could also be the reason for the slightly better image quality of PBV reconstructions, due to an increased quality of spectral separation, on the other hand. The higher tube current time product and the thereby increased radiation dose is required in the vDE protocol to account for low image noise in the low-contrast tissues of the abdomen. The high contrast structures of the lungs are rather insensitive to different radiation dose settings and the mean attenuation values are generally not affected. We therefore rate the impact of the tube current setting on the PBV evaluation as negligible. The lower rotation speed and the smaller collimation in vDE support the higher radiation dose application, but increase the scan time. However, dynamic processes during the portal venous phase are rather slow and should not affect the image evaluation. We interpret the comparable overall image quality of PBV reconstructions as proof of this assumption. Best comparability between vDE and aDE could be achieved by prospectively designed dual-phase examinations with intra-patient comparisons in future protocols, but with the drawback of an increased radiation dose for the individual.

Second, lung volumes were smaller in aDE, most likely due to shortness of breath and anxiety in the emergency setting. However, the rate of corrections for the upper segmentation threshold was comparable in both groups, indicating that slightly different volumes should not impact the PBV evaluation. Third, the clinical impact of new findings in vDE remains unclear. Prospectively designed studies should also include additional physiological measurements such as pulmonary and cardiac function. Fourth, we can only provide knowledge about one single DECT technique and one post-processing algorithm. Results obtained with other technologies or using other vendors could be different. Fifth, even though only a few patients were considered completely unevaluable because of poor PBV quality, the proportion of patients with limited image quality was roughly one fifth in both collectives (vDE 19% and aDE 21% with a Likert rating ≥ 4). Hence, robustness should be further improved in future versions through both hardware and software improvements.

In conclusion, PBV reconstructions from DECT acquisitions in the portal venous contrast phase provide robust subjective and objective image quality in the majority of patients and in comparison, to the reference in the arterial phase. The distribution ratio between the large vessels and the lung parenchyma is increased in vDE compared to aDE, suggesting an interstitial accumulation that has not yet been described before. Additional functional information about the lung parenchyma can thus be derived. Therefore, the evaluation of PBV should be considered whenever spectral datasets of the chest are acquired. Knowledge of the different image artifacts can be helpful during interpretation. Further prospective studies are recommended to evaluate potentially relevant physiological and pathological findings in venous or double-contrast phase examinations of the lungs.

## Figures and Tables

**Table 1:** Dual-energy protocols for portal–venous thoraco-abdominal CT in the study group (vDE—portal-venous thoraco-abdominal Dual Energy CT) and pulmonary arterial CT of the chest in the reference group (aDE—pulmonary arterial Dual Energy CT).

Scan Parameters	vDE Study Group (*n* = 95)		aDE Reference Group (*n* = 94)	
Tube	A	B	A	B
Tube voltage	70 kV	Sn 150 kV	80 kV	Sn 150 kV
Ref. mAs	560 mAs	140 mAs	100 mAs	77 mAs
Rotation Time	0.5 s		0.25 s	
Delay	70 s		Bolus tracking + 8 s	
CTDI	8.23 ± 2.0 mGy		5.4 ± 2.4 mGy	
DLP	588 ± 151 mGy*cm		208 ± 95 mGy*cm	
Pitch	0.6		0.6	
Collimation	128 × 0.6 mm		192 × 0.6 mm	

**Table 2:** Objective image quality of the study (vDE—portal-venous thoraco-abdominal Dual Energy CT), the reference (aDE—pulmonary arterial Dual Energy CT), and the subgroup of the reference with exclusion of cases with pulmonary embolism (aDEex): Total lung volume (VT); mean relative attenuation of the lung parenchyma (AT), ascending aorta (AA), and pulmonary trunk (AP); and distribution ratio (DL) of contrast agent between the large vessels and the lung parenchyma.

	vDE (*n* = 95)	aDE (*n* = 94)	*p*-Value	aDE_ex_ (*n* = 62)	*p*-Value
V_T in [ml]_	4795 ± 1195	3885 ± 1307	<0.01	3760 ± 1267	<0.01
A_T__in [HU]_	23 ± 6	23 ± 7	0.87	23 ± 7	0.84
A_A in [HU]_	224 ± 39	252 ± 78	<0.01	247 ± 79	0.03
A_P in [HU]_	224 ± 39	304 ± 107	<0.01	301 ± 102	<0.01
D_L in %_	10 ± 2	8 ± 2	<0.01	9 ± 2	<0.01

**Table 3:** Image artifacts in the perfusion blood volume (PBV) images: Nine different types of artifacts were found repeatedly and were classified into five groups based on their origin. Results are displayed as total number (*n*) and percentage (%) in the study (vDE—portal-venous thoraco-abdominal Dual Energy CT) or reference group (aDE—pulmonary arterial Dual Energy CT).

Artifact Group	vDE *n* = 95		aDE *n* = 94		*p*-Value
and subgroup	total cases (*n*)	relative cases (%)	total cases (*n*)	relative cases (%)	vDE vs aDE
**Streak artifacts**	**117**	**123**	**109**	**116**	**0.560**
Spine	82	86	35	37	<0.001
Contrast agent filled vessels	27	28	70	75	<0.001
Metal	8	8	4	4	0.243
**Rotation artifacts**	**121**	**127**	**125**	**133**	**0.568**
Heart	87	92	80	85	0.167
Diaphragm	34	35	45	48	0.093
**Dual Source artifacts**	**5**	**5**	**7**	**7**	**0.541**
**Recess artifacts**	**62**	**65**	**23**	**24**	**<0.001**
Anterior (costomediastinal)	45	47	21	22	<0.001
Posterior (azygoesophageal)	17	18	2	2	<0.001
**Subpleural artifacts**	**26**	**27**	**7**	**7**	**<0.001**

Bold—main group with the subgroup listed below.

**Table 4:** Amount of perfusion relevant pathological findings in total and as percentage of patients within portal venous (vDE—portal-venous thoraco-abdominal Dual Energy CT) and pulmonary arterial phase Dual Energy (aDE—pulmonary arterial Dual Energy CT).

Pathological Finding	vDE (*n* = 95)		aDE (*n* = 94)		*p*-Value
Type	Total	%	Total	%	vDE vs aDE
With anatomical correlate	11	12	39	41	0.001
Decreased vascularization	1	1	25	27	0.002
Increased vascularization	0	0	3	3	0.757
Decreased parenchyma density	4	4	3	3	0.609
Increased parenchyma density	4	4	6	6	0.306
Architectural distortion	2	2	2	2	0.999
Without anatomical correlate	7	7	12	13	0.049
Lobar	7	7	7	7	0.999
Anteroposterior gravity gradient	0	0	5	5	0.606
